# Why are older women not having surgery for breast cancer? A qualitative study

**DOI:** 10.1002/pon.3764

**Published:** 2015-02-02

**Authors:** Anne Marie Sowerbutts, Jane Griffiths, Chris Todd, Katrina Lavelle

**Affiliations:** ^1^ School of Nursing, Midwifery and Social WorkUniversity of ManchesterManchesterUK; ^2^ Manchester Academic Health Sciences Centre (MAHSC)University of ManchesterManchesterUK

## Abstract

**Objective:**

Surgery is the mainstay of treatment for breast cancer. However, there is evidence that older women are not receiving this treatment. This study explores reasons why older women are not having surgery.

**Methods:**

Twenty eight in‐depth interviews were conducted with women over 70 years old with operable breast cancer receiving primary endocrine therapy (PET) as their primary treatment. The interviews focused on their perceptions of why they were being treated with PET rather than surgery. Transcripts were analysed using the Framework method.

**Results:**

Based on reasons for PET, patients were divided into three groups: ‘Patient Declined’, ‘Patient Considered’ or ‘Surgeon Decided’. The first group ‘Patient Declined’ absolutely ruled out surgery to treat their breast cancer. These patients were not interested in maximising their survival and rejected surgery citing their age or concerns about impact of treatment on their level of functioning. The second group ‘Patient Considered’ considered surgery but chose to have PET most specifying if PET failed then they could have the operation. Patients viewed this as offering them two options of treatment. The third group ‘Surgeon Decided’ was started by the surgeon on PET. These patients had comorbidities and in most cases the surgeon asserted that the comorbidities were incompatible with surgery.

**Conclusions:**

Older women represent a diverse group and have multifaceted reasons for foregoing surgery. Discussions about breast cancer treatment should be patient centred and adapted to differing patient priorities. © 2015 The Authors. *Psycho‐Oncology* published by John Wiley & Sons Ltd.

## Introduction

Breast cancer is the most common cancer in women in England with incidence rates of 222.9 per 100 000 for 45–49 years old rising to 439 per 100 000 in those aged 90 years and older 1. Surgery is the mainstay of treatment for operable breast cancer 2. UK national guidelines stipulate that patients of all ages with early‐stage breast cancer should be offered surgery unless precluded by significant comorbidity and following surgery adjuvant treatment (radiotherapy or chemotherapy) should be considered 3. There is evidence that older women are not receiving surgery 4, 5, 6, 7, although relatively little is known about the underlying reasons for this.

If women with breast cancer do not receive surgery, then they are usually treated with primary endocrine therapy (PET); this treatment can become ineffective at controlling disease after a period of time 8. There has been some research investigating surgeons' reasons for using PET rather than surgery. The predominant reasons cited by surgeons were patients being unfit for surgery, comorbidity, patient preference and old age 9, 10. However, these studies are from the surgeon's perspective, and there is little research from the patient's viewpoint.

In particular, there has been insufficient research into reasons why patients choose not to have surgery. Lally examined decision making between different types of surgery and found that older women wanted more information on surgical recovery and maintaining independence, so one could speculate that these reasons are also important for women deciding whether or not to have surgery 11. However, this was not borne out in a study by Husain *et al*. 12. They explored treatment choices with 21 women over 70 years old who were offered the option of surgery or PET. They found the main reason women opted for PET was doctor's advice. Other reasons were PET was a worthwhile first option with surgery as backup treatment and a wish to avoid further interventions after painful biopsies. A limitation of this study was that interviews took place up to 15 years after diagnosis. Thus, the mere fact of survival may have influenced women's views on their treatment, and the long period since treatment may have compromised recall. Moreover, clinical practice and societal attitudes have changed in the intervening years with the emphasis on offering surgery rather than PET 4.

We recently reported on a quantitative study investigating reasons for the lack of surgery for older breast cancer patients 5. However, the scale of this study necessitated use of a broad measure of patient choice based on responsibility for treatment decision. We therefore incorporated a nested qualitative study to explore with women in detail factors influencing a decision not to have surgery when that occurred.

## Methods

This study was a qualitative nested component of a larger quantitative project examining the role of patient health and choice in the surgical treatment decision in women aged ≥70 years with operable invasive breast cancer. Fuller methods, inclusion and exclusion criteria are described elsewhere 5. In brief, 800 women predominantly from North West England took part within 30 days of diagnosis in a quantitative survey about health‐related quality of life and treatment decision measured by the control preference scale 13, 14. This was followed by case note review at 3 months post‐diagnosis; data were collected on tumour characteristics and staging at diagnosis, treatment and comorbidities.

In the qualitative study reported here, women who did not have surgery were asked open‐ended questions to ascertain reasons for that treatment decision. Interviews occurred in the woman's home within 30 days of diagnosis and occasionally, with patient consent, family members sat in on the interviews. This paper reports on the findings of 28 women who were treated with PET and recruited after ethical approval for the nested study was obtained. Whilst this was all the patients available to enter the study, sample size in qualitative research is rooted in saturation of themes, which typically occurs around 30 patients 15.

### Sample

The age range of the 28 women was 76–99 years (mean 86 years) with most women aged over 80 years (Table 1). The women were predominantly professional/intermediate social class, white ethnic group and living in North West England 16. All patients were oestrogen‐receptor positive therefore likely to be responsive to PET, which is only effective in oestrogen‐receptive positive tumours 8.

**Table 1:** Patient age and medication by decisional group

Patient number	Age (years)	Medication	Group
1	90	Letrozole	Patient declined
2	83	Letrozole	
3	90	Letrozole	
4	86	Letrozole	
5	87	Letrozole	
6	87	Letrozole	
7	92	Tamoxifen	
8	91	Letrozole	
9	92	Letrozole	
10	99	Tamoxifen	
11	90	Letrozole	Patient considered
12	84	Letrozole	
13	90	Letrozole	
14	85	Letrozole	
15	85	Tamoxifen	
16	85	Arimedex	
17	91	Letrozole	
18	85	Letrozole	
19	85	Letrozole	
20	77	Letrozole	Surgeon decided
21	91	Letrozole	
22	80	Letrozole	
23	89	Letrozole	
24	90	Letrozole	
25	81	Letrozole	
26	76	Letrozole	
27	78	Tamoxifen	
28	78	Letrozole	

### Data collection

We used an approach common to many qualitative studies 17, 18, 19. Interviewers (AMS or KL) used a topic guide to direct the interview. However, spontaneous narratives were also encouraged. Each qualitative interview started with the woman being asked an open‐ended question to help recall her cancer journey from finding the lump until making the treatment decision. Interviewees were then probed further on treatment options and information given by the surgeon. They were also asked about who was responsible for the treatment decision and reasons why the particular treatment decision had been made. Patients were interviewed once, and interviewers were with the women for 40–90 minutes. All interviews were audio recorded and transcribed verbatim.

### Analysis

Data were analysed using NVivo10 20 and the Framework method 21. The Framework method is a frequently used form of qualitative content analysis, which follows a five‐step process 21. 1) Familiarisation: all transcripts were read and reread to become familiar with them. 2) Identifying a thematic framework: a thematic framework was identified from reading the transcripts, proposed by AMS and modified during discussion with the rest of the team. 3) Indexing/coding: AMS coded all the manuscripts based on the agreed thematic framework. 4) Charting: AMS produced thematic charts from this coding and indexing. Coding and thematic charts were discussed and agreed by AMS, KL and JG. 5) Mapping and data interpretation: the whole team was involved in this process. The analysis presented is based on team consensus of the interpretation of the transcripts.

## Results

All patients were on PET but varied on whether they or the surgeon made the treatment decision. Analysing the patient interviews using the Framework method, the patients were divided into three decisional groups (Table 1). The first group ‘Patient Declined’ absolutely ruled out surgery stating they did not want an operation. The second group ‘Patient Considered’ comprised women who considered treatment options of surgery or PET and opted for PET. The third group ‘Surgeon Decided’ consisted of patients who were started by the surgeon on PET. All the patients in this third group had comorbidities, and in most cases, the surgeon asserted that the comorbidities were incompatible with undergoing surgery.

### 1. Patient declined surgery

These patients were unequivocal that they did not want surgery for their breast cancer.

#### Perceptions about age

All patients in this group were over 80 and most over 85 years old. Most patients cited their age as a reason for not having surgery, for example, ‘At 91 I think I could do without surgery’ (PT8), but many said they would have had the operation if they had been younger. However, there seemed to be various motivations behind women identifying themselves as too old for an operation. Some patients were not interested in prolonging their life; they felt they had lived their life, and there was nothing left that they wanted to do:

I don't want an operation at my time of life really…I mean what is there, I'm just sat there looking through the window in there, I've got the telly and I've got books and…you get tired, you get that you've done it all, said it all (PT6).

Others commented that they had various health problems so it made no sense to them to have an operation to gain more years of a restricted life. Other patients spoke about having a limited life span and not having much longer to live and questioned what difference having an operation would make, as one patient said, ‘Something's going to get you. So what is the good of prolonging it when you get to this age?’ (PT2). However, although at their age they were not keen on prolonging their lives, patients did want to retain their current level of independence, as one patient said ‘[its] very important that I keep not depending on people, I know I depend on shopping and all that but such as changing and taking me to the toilet,’ (PT6).

#### Attitude to diagnosis

Some patients were unconcerned about their diagnosis, and this attitude seemed to be linked to not wanting aggressive treatment. One patient said about PET, ‘But to me if it's not successful so what. I'll go with something eventually won't I?’ (PT9). When asked if she would think about an operation if the tablets were not successful, she replied emphatically ‘No, no, no.’ When these patients mentioned the diagnosis, they said that they were not worried about it. One woman said ‘I would have been just as worried if you told me I had a cold’ (PT5) and another commented ‘In fact you know, it's a funny thing, but it doesn't really worry me…no, I just accepted it. May be that is a good thing. I've got to go some way, so I may as well go this way’ (PT10).

#### Attitude to operations/hospital stay

Additionally, some women had concerns about aspects of surgery or going into hospital either because of comorbidities or because of the experience of others. Surgery was only seen negatively; patients did not consider whether surgery could improve the quality of their lives. In contrast, PET was seen as an option that would enable them to avoid hospital and treatments that would make them feel ill. The women reported that the surgeons accepted their decision; the surgeons did not try to persuade them to have surgery nor did they discuss the possibility that the tablets may cease to be effective. As one patient said ‘The doctor…said, “You've made your mind up, we'll not alter it.” I said, “No, you won't” ’ (PT3).

### 2. Patient considered surgery

Having been offered a choice between surgery and PET, these patients weighed up the options before deciding on PET.

#### Surgery as a fallback option

The majority of patients who considered both treatments decided on PET as this did not rule out future surgery. They viewed this as giving them two choices. They said taking PET first would allow them to see if it could be effective and if it did not work they would have the operation. ‘Yes, because you've got two choices then haven't you? If the first thing fails then perhaps do the operation’ (PT14).

Surgeons also seemed to promote tablets as offering the option of surgery later. As one patient said, ‘[the surgeon] said, you can always have the operation at a later date, if you feel so inclined, you've only got to say so’ (PT12). What is not clear is the information surgeons gave about tablets becoming ineffective. Some patients or relatives did say that the surgeon said that if one tablet became ineffective, another could be tried. However, no patients described being told that all tablets may become ineffective and in this case an operation would be the only effective treatment.

#### Adjuvant treatment

For some patients concerns about adjuvant treatment prompted them to opt for PET. One patient was under the impression that follow‐up treatment was always necessary after surgery. She commented, ‘It's not the surgery I was keen to avoid, it's the two or three times a week treatment that you have to endure’ (PT19), and she would have considered surgery if this could have been avoided. Another patient had been told that lumpectomy and radiotherapy would be the optimum treatment but was concerned that radiotherapy would exacerbate an existing skin problem. She commented that she had thought about lumpectomy, but said ‘if it's followed by radiotherapy, the burning of my skin, you see…so I had to take that into account, because my skin would be really affected.’ (PT20).

#### After effects of operation

In other cases, patients or relatives concerns about the after‐effects of the operation prompted their choice for PET. One relative said that his mother would not be able to cope with keeping the bandage dry and he had concerns about infections. In two other cases, concerns were raised about lymphoedema.

#### Influence of wider family

In two cases, the patient's family were instrumental in the treatment decision, discussing in detail risks and after‐effects of the operation with the medical team and concluded that surgical treatment would not be right. However, in both cases, although the patient did not examine the risks and benefits in detail, they both said they would prefer not to have surgery.

### 3. Surgeon decided against surgery

For some patients, the surgeon decided they should be treated with PET. In all cases, the patient mentioned having comorbidities. In addition, several patients were told they were unfit for general anaesthetic; as one patient said, ‘I could not have [general anaesthetic] because it affects my heart, you see’ (PT23).

Three patients perceived that the choice of surgery or PET was discussed with them, but the doctor made the treatment decision. Other patients were not given a choice of treatments. However, all patients accepted the doctor's decision that they should have PET, mostly without question. Only one patient, judged unfit for general anaesthetic, questioned the decision, enquiring about the possibility of using an epidural and was told this was not possible ‘because it only goes down [the body] not up’ (PT28). Local anaesthetic, which may be used when general anaesthetic is not recommended, did not seem to be discussed.

The treatment decision seems to have been driven by the doctor's recommendation rather than patient's psychosocial beliefs or attitude to diagnosis. No one said that they had had enough of life and two patients talked about wanting to live a long time. Although some were shocked about finding the lump, they did not delay seeking medical treatment. A number of patients in this category expressed worry, upset or fear about the diagnosis. One patient said ‘I know I've been told it's not life threatening and I'm not to worry, but you can't help it’ (PT20). Another said ‘I'm very upset about it to be honest…very upset’ (PT27).

## Discussion

Surgery is the recommended first line of treatment for early breast cancer for women of all ages, and this is reflected in national guidelines 2, 3. However, the patients in this study were not having surgery and were being treated with PET. Reasons participants gave for being on PET rather than having surgery varied and can be categorised into three broad groups. In the first group, ‘Patient Declined’, patients were not interested in undergoing surgery either because they wanted to avoid treatments that would impact on their current fitness level or because of beliefs about their age. These patients, who tended to be older, mentioned their age, which seemed to stand as a shorthand for a diversity of reasons: having a limited life span, not wanting to prolong their life either because of comorbidities or having nothing left to do. Patients in the second group, ‘Patient Considered’, considered the possibility of surgery but opted for PET in the belief that they could have surgery later if tablets failed or they wanted to avoid the after‐effects from more aggressive treatments. Patients in the third group, ‘Surgeon Decided’, were started by the surgeon on PET. All patients in this group had comorbidities and in some cases the surgeon said to them explicitly that surgery was not possible because of their health.

Few studies have investigated reasons why breast cancer patients opt for PET instead of surgery, Husain *et al*. is a notable exception 12. Husain *et al*. examined reasons for treatment decisions in women over 70 years old offered surgery or PET. They found that women followed the doctor's recommendation and did not mention age in making their decision; as such they were most like our ‘Surgeon Decided’ group. In contrast, women from our ‘Patient Declined’ group did mention age as a motivation for ruling out surgery. Patients used their age as an indicator that they were at the end of their life. Whilst most patients did not want an immediate end, they were not interested in prolonging their lives as long as possible and in some cases did not want the quality of their life affected by surgical treatment and therefore declined surgery. What patients seemed to be articulating in discussing their lives is what Morrison refers to as having a sense of completeness that life has run its course 22.

Viewing themselves at the end of their lives led women in the ‘Patient Declined’ group to question what difference surgery would make. The thinking behind this seemed to be that life for them was limited so although the cancer may be removed, because of their age, they would develop another disease. This interpretation was reinforced by several patients saying that they would have had the operation if they were younger. Similarly, Sinding *et al*. found some women over 70 years old with cancer foregoing treatment because of their age but commenting that treatment would have been pursued at a younger age 23. In contrast, in patients aged 65‐74 years, Newcomb *et al*. did not observe rejection of treatment for the reason of fatalism about their advancing age; however, the patients in their study were a lot younger 24. Yoo *et al*. investigating patients 65‐82 years old found some patients mentioned not being concerned about dying and expecting to die soon but did not discuss this as leading them to forego treatments 25.

Comparing the ‘Patient Declined’ with the ‘Surgeon Decided’ groups; the patients from these two groups had very different attitudes to their cancer diagnosis. For the ‘Patient Declined’ group, this seemed to be an influence on their treatment decision. These patients viewed their lives as at an end and seemed unconcerned about their cancer diagnosis with many saying that they were not worried. Some of the patients even discussed their cancer as potentially the way they would die, although all the patients had early‐stage disease. They were not interested in pursuing aggressive treatments to prolong their lives and rejected surgery. This is in sharp contrast with patients in the ‘Surgeon Decided’ group. Many of these patients were worried about the cancer diagnosis, and no patients discussed their lives as being complete. In fact, two patients explicitly said they wanted to live a long time. These patients were not offered any treatment other than PET. Research has found older patients who are anxious or want to prolong their life as much as possible want more treatment; therefore, it is possible that had these patients been offered more aggressive treatment they would have opted for it 26, 27.

Women in the ‘Patient Considered’ group weighed up the possibilities and opted for PET. They chose PET as if this did not work, then they understood they still had the option of surgery. They viewed this as giving them two choices; Husain *et al*. found patients giving similar reasoning in their study. However, what patients did not discuss in the interview is that if PET failed they would be facing an operation at an older age when comorbidities (which increase with age 28, 29) may be worse. Patients were on aromatase inhibitors or tamoxifen, and a meta‐analysis has found the rates of disease progression as 31% versus 46%, respectively 30. When progression occurs, patients need to change to an alternative hormone therapy or undergo an operation. It is not clear what information patients had and understood about the effectiveness of tablets.

Giving clear information on the effectiveness of PET is problematic as there are gaps in the evidence base. Elderly patients are underrepresented in clinical trials, and trials aimed at elderly patients have closed because of lack of recruitment; notable in this context is the Esteem trial, which was comparing aromatase inhibitors with surgery 31, 32, 33. There is evidence that surgeons are more likely to offer choice when the evidence base for treatments is uncertain 34. This may in part explain why surgeons offered patients a choice of PET or surgery. Therefore, further studies aimed at the impact of treatment on elderly breast cancer patients are warranted 35.

### Limitations

First, all patients interviewed either rejected surgery or were started by the doctor on PET possibly on the basis of their comorbidities. However, this was just a subset of patients in the larger study. The overall sample for the most part was made up of patients who underwent surgery but also contained a larger proportion of patients being treated with PET who left the decision up to the surgeon. In the larger quantitative study, patient choice, as measured by the Control Preferences Scale 13 was not found to explain why patients were treated with PET. A second limitation was, as the study progressed, family members were found to be important influences in treatment decision making. Occasionally, with patient permission, relatives sat in on interviews. However, this occurred on an impromptu basis, and further systematic investigation of the influence of relatives on the treatment decision would be warranted. Another limitation is all the women identified themselves as white British and ethnic minority women may have differing views and needs 36, 37.

### Summary and future directions

Despite these limitations, this study shows the benefit of qualitative research in exploring decision making. It offers new ways of examining treatment choices of older women providing further understanding on reasons why they are not having surgical treatment for early‐stage breast cancer. In the ‘Patient Declined’ group, their reasons were linked to their lack of desire to prolong their lives at the expense of undergoing surgery and lack of worry about their cancer diagnosis. This can be contrasted with the ‘Surgeon Decided’ group in which many of the women expressed worry about the cancer diagnosis and were keen to prolong their lives. However, the surgeon took the treatment decision for PET possibly because of concerns about comorbidities. The ‘Patient Considered’ group weighed up the options presented and opted for PET as a first option, with the understanding that surgery could be subsequently performed if PET failed. However, the research to inform this decision is lacking, and further research is warranted.

The elderly breast cancer population is widely acknowledged as a diverse group 38. This was reflected here with patients having differing priorities and reasons for being on PET; therefore, patient‐centred care is crucial in this group.

## Conflict of interest

The authors have declared no conflicts of interest.
